# Ambient lighting alters pattern electroretinogram P50 peak time and spatial sensitivity

**DOI:** 10.1007/s10633-024-09984-9

**Published:** 2024-08-14

**Authors:** Lisa Tucker, Oliver R. Marmoy, Siân E. Handley, Dorothy A. Thompson

**Affiliations:** 1 Clinical and Academic Department of Ophthalmology, Tony Kriss Visual Electrophysiology Unit, Great Ormond Street Hospital for Children NHS Foundation Trust, 40-41 Queens Square, London, UK; 2 UCL Great Ormond Street Institute of Child Health, University College London, 30 Guildford Street, London, UK

**Keywords:** Pattern ERG, PERG, Room light, Ambient background light, Contrast, Luminance, Spatial sensitivity

## Abstract

**Purpose:**

Our aim was to explore the effect of ambient lighting on the pattern ERG (PERG).

**Methods:**

We compared PERGs recorded in two conditions; room lights on and room lights off. PERGs from 21 adult participants were recorded from each eye to high contrast checks of 50’ side width, reversing 3rps in a large (30°) and then standard (15°) field. This was performed first in lights-ON conditions, then 2 min after the room lights were switched off. A minimum of 2 averages of 300 trials were acquired for each condition. A subset of 10 participants had PERGs recorded to a 50’ check width with a range of stimulus contrasts (96–18%), also to a range of different check widths (100’–12’) at high contrast in both ambient lighting conditions in a 30° field.

**Results:**

The lights-ON P50 median peak time (PT) was 3 ms earlier than the lights-OFF P50 from the 30° field (range 0–5 ms) and 15° field (range 0–6 ms). The earlier lights-ON P50 PT was evident at different stimulus contrasts, even after accounting for stimulus contrast reductions associated with stray ambient lighting in lights-ON conditions. Lights-OFF and lights-ON P50 PT were similar to different check widths; the lights-OFF P50 PT to a 50’ check width matched the lights-ON P50 PT to a 25’ check width.

**Conclusion:**

PERG P50 PT in lights-ON ambient light conditions can be earlier than in lights-OFF ambient light conditions. The difference in P50 PT with ambient light may reflect alterations in spatial sensitivity associated with retinal adaptation. These results emphasise the clinical importance of consistent ambient lighting for PERG recording and calibration.

## Introduction

The pattern electroretinogram (PERG) is a localised test of central retinal function. The waveform of the PERG is typically triphasic characterised by two major components: the P50 and N95. The original work of Luo and Frishman [1] demonstrated the dominance of retinal ganglion cell (RGC) contribution to the generation of the PERG waveform. Although the P50 is driven by macular cones, it is estimated that 70% of the P50 and 100% of the N95 are generated by the inner retina, in particular RGCs [2–4].

Clinically the P50 is an important bridge that distinguishes maculopathy from primary RGC disease or optic neuropathy as a cause of an abnormal pattern visual evoked potential (VEP) [5]. In patients with maculopathy, the P50 amplitude diminishes with a proportional reduction of N95 amplitude and/or P50 PT becomes prolonged [5]. In RGC disease such as LHON or optic neuropathy the N95 amplitude can be attenuated selectively, the N95:P50 amplitude ratio reduces, and/or P50 PT can shorten [5–9].

The major studies of how the PERG varies with alteration in contrast or luminance took place in the decade 1980–1990 [10–17]. These studies informed the specifications of subsequent clinical guidelines and Standards. The International Society for Clinical Electrophysiology of Vision (ISCEV) PERG Standard 2012 [18] suggested that background room lighting is not critical. During discussions in the ISCEV technical courses it was highlighted that a wide range of ambient lighting is used during clinical Standard PERG recordings internationally. Some laboratories use photopic ambient lighting reasoning that the PERG is a cone driven response which should be enhanced with room lights on, whilst others, as in our centre, carry out PERGs with room lights off to minimise distractions for children.

With such diversity of practice, we sought to understand the effects of ambient lighting on the PERG. In this study we compared the ISCEV Standard PERG from both 30° and 15° fields under lights-ON and lights-OFF ambient lighting recorded in the same participants. We also explored the effects of stimulus contrast and check width in these ambient light conditions to evaluate the spatial sensitivity changes (i.e. the spatial tuning of the PERG with check width alteration).

## Methods

### Participants

We recruited 21 adult participants (17 female, 4 male) with a median age of 31 years (range 23–51 years) with no ophthalmological concerns and best corrected visual acuity with either eye better than or equal to 0.00 LogMAR. Participants with refractive errors greater than ± 7.00DS were excluded. Iris colour was recorded as ‘light’ (blue/green/hazel) or ‘dark’ (brown).

### PERG stimulus and recording

**Fig. 1:**
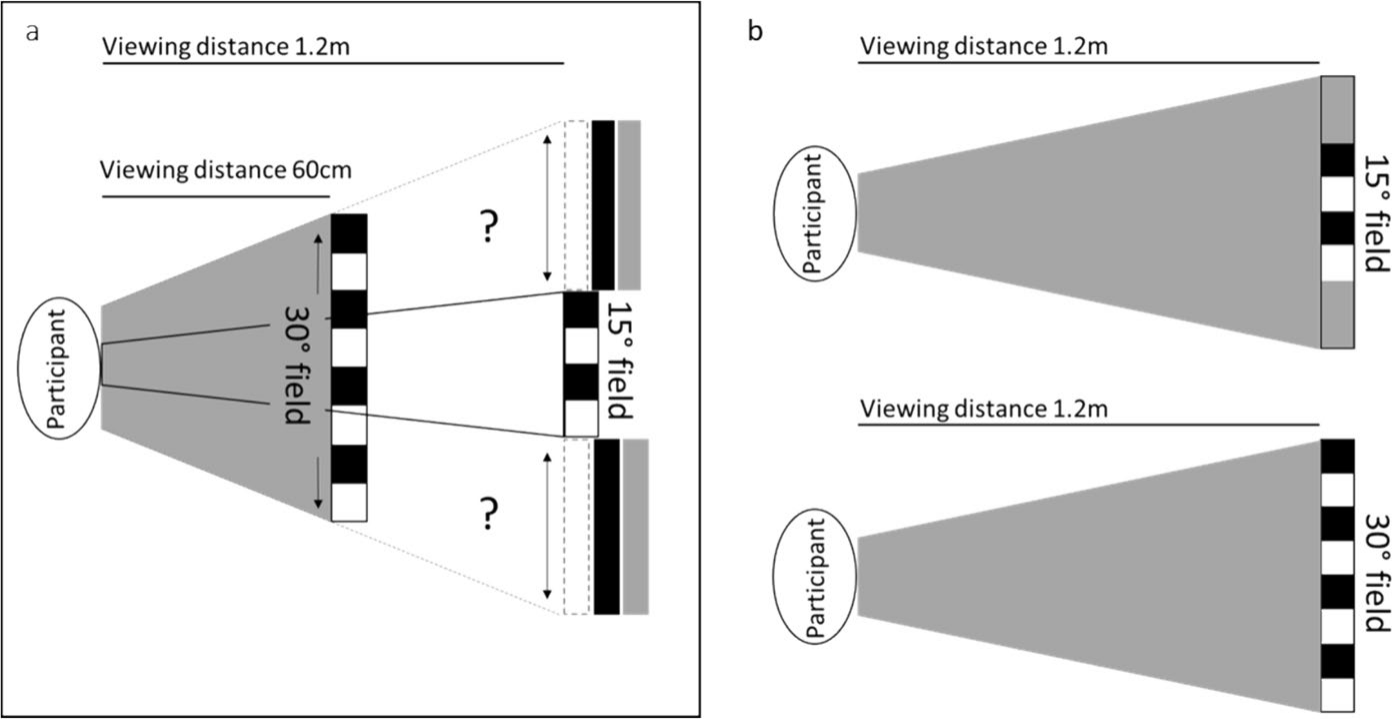
Possible changes of stimulus surround luminance with viewing distance. **a** Schematic showing how surround luminance may change depending on the background surround of the stimulus monitor, should the monitor need to be moved closer to achieve a larger field size. The ‘?’ highlights the uncertain surround background luminance which may be lighter, darker, or matched to the mean stimulus luminance. **b** Schematic to illustrate how surround luminance was maintained for the two field sizes through the mean surround luminance of a large stimulus (PDP) monitor

**Fig. 2:**
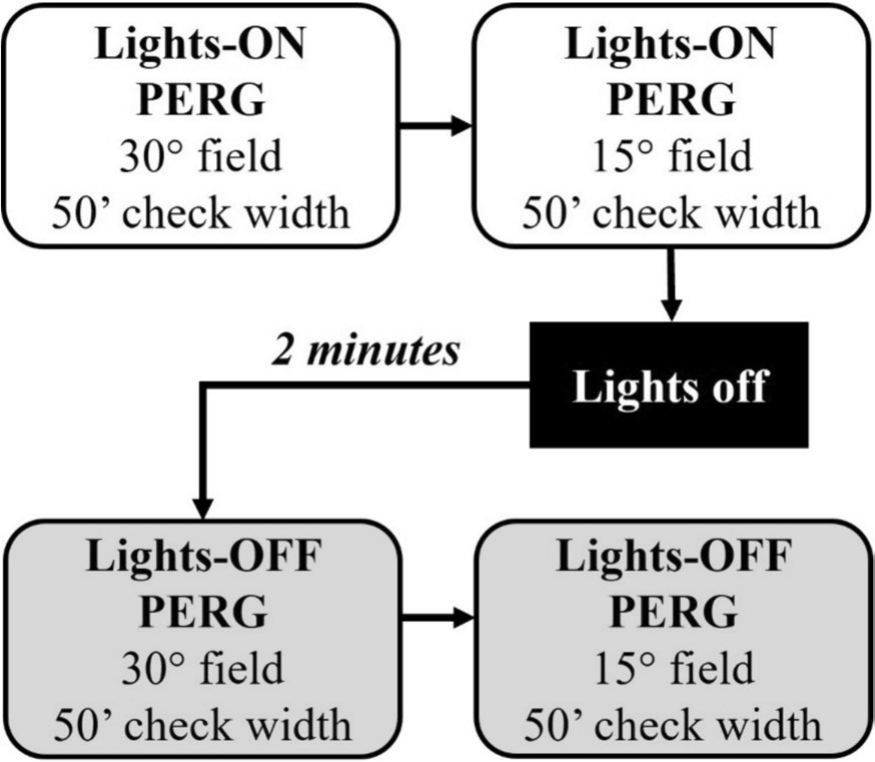
Procedural steps for participant recording in this study

PERGs were recorded using corneal fibre electrodes (Sterile ERG Thread Electrode, Unimed Electrode Supplies Ltd.) referred to skin electrodes (Disposable 4-disk electrodes, natus neurology incorporated) on the ipsilateral outer canthus using a diagnosys espion E3 system. Bandpass filtering was set to 0.3–100 Hz with an automatic rejection enabled for signals exceeding ± 100 µV over the whole sweep range of 300 ms, including a 15 ms pre-stimulus interval. A minimum of 2 averages of 300 trials were acquired for each PERG trace. High contrast black and white checks of 50’ side width, were presented at 3 rps in 15° and 30° fields on a plasma display panel (PDP) viewed at 1.2 m with a mean surround luminance (Fig. 1). Pupil size and fixation was monitored using an infrared camera and data acquisition was paused if fixation was lost. First, a PERG was recorded with room lights on, followed by a PERG recorded two minutes after the room lights were switched off (Fig. 2). Ten participants also had 30° field PERGs recorded in lights-ON and lights-OFF conditions to (i) a 50’ check width presented at measured contrasts ranging from 96 to 18%, and (ii) at the highest measured contrast to a range of check widths (100’, 50’, 25’ and 12.5’).

### Statistical analysis

The amplitudes and PTs of the PERG P50 and N95 components were measured [9]. Interocular symmetry was assessed using a Pearsons correlation coefficient. A Shapiro–Wilk test demonstrated a non-normal distribution of these data and non-parametric statistics were therefore used for all analyses. PERG P50 amplitude and PT, N95 amplitude and N95:P50 amplitude ratio were compared between lights-ON and lights-OFF conditions using a Wilcoxon signed rank test. PERG P50 amplitude and PT were plotted to a range of stimulus contrasts and check widths for lights-ON and lights-OFF conditions to visualise trends. All statistical analyses were carried out using GraphPad Prism (V5). Comparative statistics for PERG P50 amplitude, P50 PT, N95 amplitude and N95:P50 amplitude ratio were corrected for multiple comparisons using a Bonferroni correction. The stated *p*-values below are those following correction.

### Photometry

The luminance of the checkerboard elements were measured at the viewing distance of 1.2 m from the screen with a calibrated spot photometer (Konica Minolta LS-110). The Michelson contrast of the checkerboard stimuli were given by ((L_max_ – L_min_)/(L_max_ + L_min_)) and mean luminance calculated as (L_max_ + L_min_)/2 [19], where L_max_ = maximal luminance of ‘white’ checks, and L_min_ = minimal luminance of ‘black’ checks. The ambient light level was measured by placing a piece of white paper next to the participant’s face and measuring its luminance under lights-ON and lights-OFF conditions.

## Results

### Photometry

The ambient light level measured 117 photopic cd.m^2^ in lights-ON conditions compared to 4 photopic cd.m^2^ in lights-OFF conditions. The luminance of the ‘black’ and ‘white’ checks measured photometrically at the manufacturer’s software maximal stimulus contrast setting (100%) gave actual Michelson contrast under lights-ON conditions of 85.3% with a mean luminance of 78.8 cd.m^2^, and under lights-OFF conditions 95.7% contrast with a mean luminance of 70.3 cd.m^2^. This reduction in calculated Michelson contrast in lights-ON conditions was due to the increased luminance of the black checks. At the maximal software contrast setting there was a difference of 10.4% stimulus contrast and 8.5 cd.m^2^ luminance between lights-ON and lights-OFF conditions. These measurements were repeated for a range of software contrast settings as shown in Table 1.

**Table 1:** Photometric measurements of the stimulus contrast and luminance in lights-ON and lights-OFF conditions

Lights-ON (cd.m^2^)		Mean luminance (cd.m^2^)	Calculated contrast (%)	Software contrast (%)	Calculated contrast (%)	Mean luminance (cd.m^2^)	Lights-OFF (cd.m^2^)	
White	Black						White	Black
73.7	62.1	67.9	8.6	10	10.5	60.6	67.0	54.3
88.8	62.2	75.5	17.6	20	20.6	66.4	80.0	52.7
96.4	55.2	75.8	27.1	30	29.8	66.8	86.7	47.0
103.4	49.3	76.3	35.4	40	39.9	67.3	94.1	40.5
117.8	37.3	77.5	52.0	60	59.0	69.5	110.5	28.4
130.8	25.2	78.0	67.7	80	76.8	69.5	122.8	16.1
145.9	11.6	78.8	85.3	100	95.7	70.3	137.6	3.0

### Inter-ocular symmetry

Right and left eye PERGs showed very strong positive correlations for P50 amplitude (r (80) = 0.95, *p* =  < 0.000), P50 PT (r (80) = 0.87, *p* =  < 0.000) and N95 amplitude (r (80) = 0.94, *p* =  < 0.000). There was a lower but still significant positive correlation between eyes for N95:P50 amplitude ratio (r (80) = 0.62, *p* =  < 0.000). Therefore, there was no advantage in including data from each eye so data from the right eye (RE) was used for all further analyses.

### P50 peak time changes with ambient lighting

The RE data from 21 participants were analysed using a Wilcoxon signed rank test. P50 PT was significantly earlier in lights-ON compared to lights-OFF conditions; with lights-ON/lights-OFF medians of 46 ms (IQR 45-48 ms)/49 ms (IQR 48-50 ms) from the 30° field (*p* < 0.0008), and lights-ON/lights-OFF medians of 46 ms (IQR 44.5-48 ms)/50 ms (IQR 47.5–50.5 ms) from the 15° field (*p* < 0.004). These measurements are tabulated in Table 2.

**Table 2:** Tabulated peak time and amplitude measures for PERGs in lights-ON and lights-OFF ambient lighting

Participants *n* = 21	30° Field lights-ON	30° Field lights-OFF	15° Field lights-ON	15° Field lights-OFF
P50 Peak time (ms)	46 (*44–49*)	49 (*45–51*)	46 (*43–52*)	50 (*46–52*)
P50 Amplitude (µV)	6.9 (*3.8–9.7*)	8.2 (*5.2–11.0*)	2.4 (*1.3–4.1*)	3.3 (*1.8–4.7*)
N95 Amplitude (µV)	10.2 (*5.0–14.0*)	10.6 (*6.0–16.0*)	4.3 (*2.9–7.3*)	5.0 (*3.2–6.6*)
N95:P50 Amplitude ratio	1.4 (*1.1–2.0*)	1.2 (*1.1–2.2*)	1.7 (*1.1–2.5*)	1.6 (*1.2–2.6*)

For each PERG measure, the median (*and range*) are shown for each field size and ambient lighting condition

### P50 and N95 amplitude changes with ambient lighting

#### For the 30° field:

P50 amplitude from the 30° field was slightly but significantly smaller in lights-ON conditions than in lights-OFF conditions with a median difference of 1.3 µV (medians 6.9 µV (IQR 6.6–7.7 µV)/8.2 µV (IQR 6.3–9.0 µV); *p* = 0.0136). N95 amplitude was not significantly different between the two ambient light conditions (medians 10.2 µV (IQR 9.0–11.2 µV)/10.6 µV (IQR 9.5–12.2 µV); *p* = 1.2328). The ratio of N95 to P50 amplitudes was significantly larger in lights-ON than lights-OFF conditions with a median difference of 0.2 (medians 1.4 (IQR 1.3–1.8)/1.2(IQR 1.2–1.5); *p* = 0.0312).

#### For the 15° field:

**Fig. 3:**
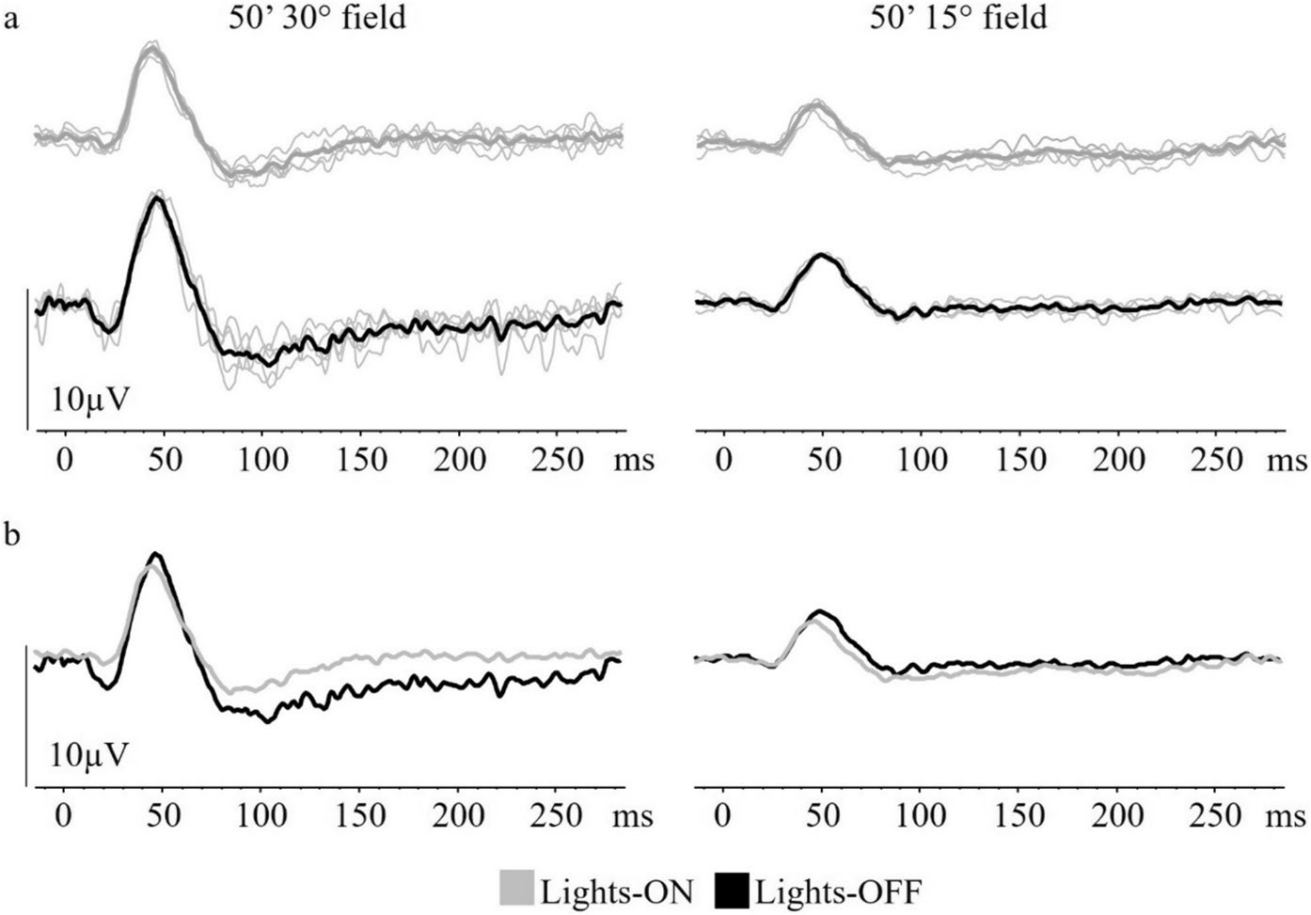
Example of lights-ON and lights-OFF PERGs from one participant from each field size. **a** The left panels illustrate the 30° field PERG and those on the right the 15° field PERG. Lights-ON PERG waveforms are shown in grey and lights-OFF PERG waveforms are shown in black. **b** PERG traces are superimposed to illustrate the waveform change between ambient light conditions

**Fig. 4:**
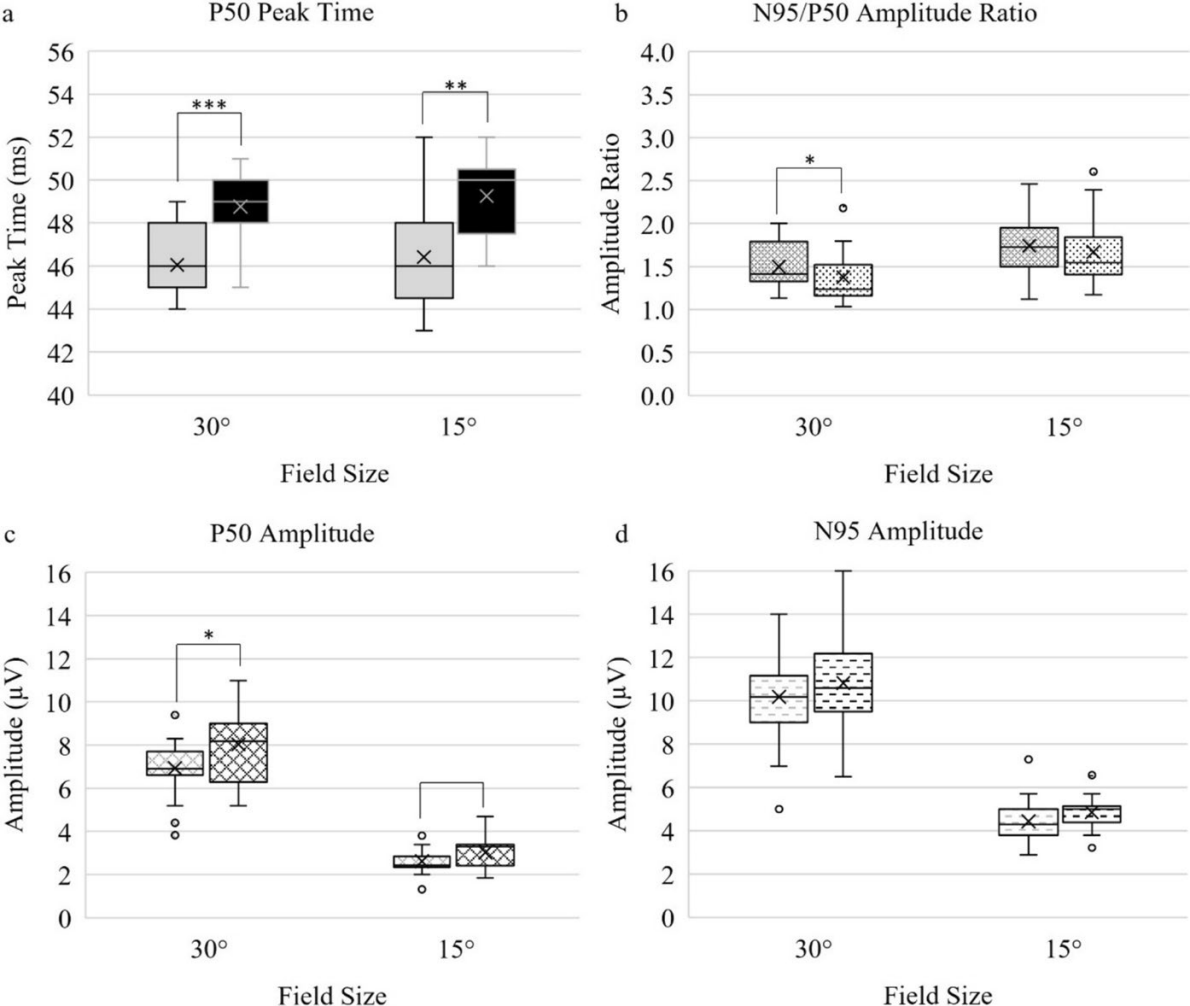
Box and whisker plots summarising the distribution of the PERG measurements in lights-ON and lights-OFF ambient light conditions. For all panels, lights-ON group data are illustrated on the left and lights-OFF data on the right. These are plotted as median values (horizontal line), mean (cross), interquartile range (25^th^–75th centile, box) and non-outlier values (whiskers). Significance between ambient light conditions is specified as *p < 0.05, **p =  < 0.01, ***p =  < 0.001. Panel A: P50 PT, Panel B: N95/P50 amplitude ratio, Panel C: P50 amplitude, Panel D: N95 amplitude

P50 amplitude from the 15° field was also slightly but not significantly smaller in lights-ON conditions compared to lights-OFF conditions with a median difference of 0.9 µV (medians 2.4 µV (IQR 2.4–2.9 µV)/3.3 µV (IQR 2.4–3.4 µV); *p* = 0.3704). N95 amplitude showed no significant difference in lights-ON compared to lights-OFF conditions (medians 4.3 µV (IQR 3.8–5.0 µV)/5.0 µV (IQR 4.4–5.2 µV); *p* = 0.1728). The ratio of N95 to P50 amplitudes did not show any significant differences with ambient light conditions (medians 1.7 (IQR 1.5–2.0)/1.6 (IQR1.4–1.8); *p* = 4.6888). PERG amplitude and PTs between ambient lighting conditions from both field sizes are summarised in Table 2. Example PERGs from one participant recorded in different ambient lighting conditions are shown in Fig. 3 and the group data in Fig. 4.

### P50 peak time changes with stimulus contrast and luminance

**Fig. 5:**
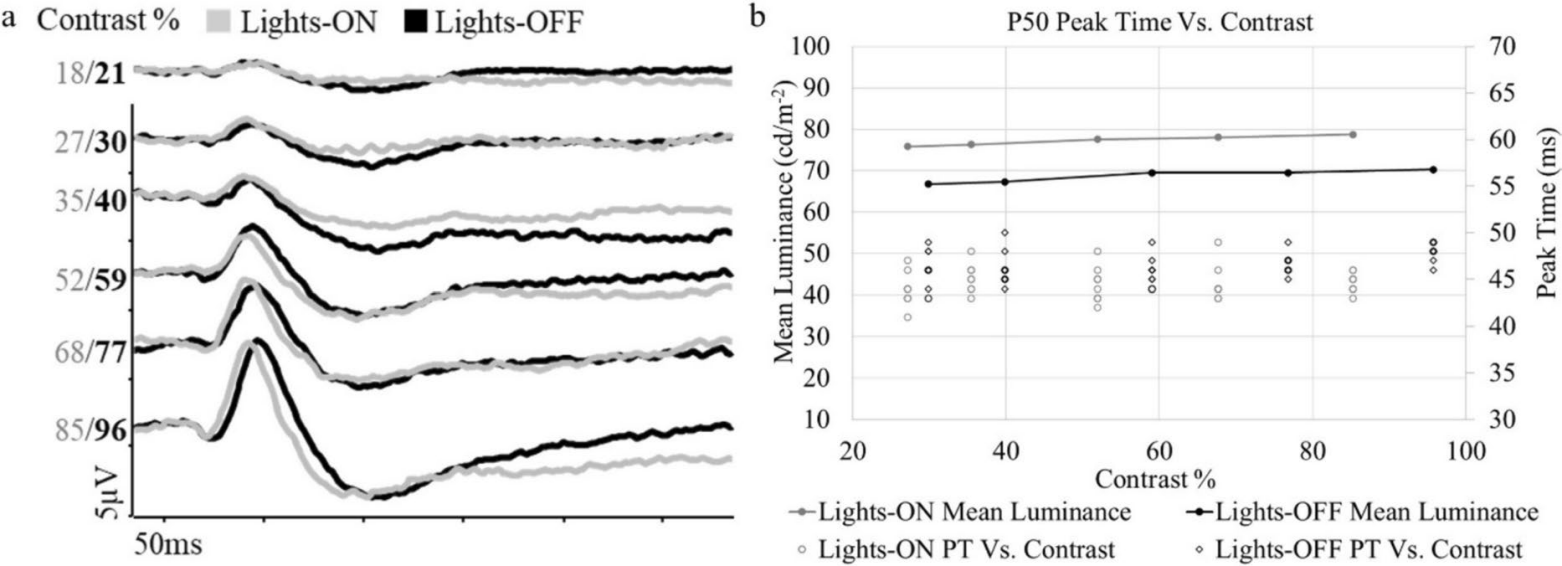
PERG changes with stimulus contrast and mean luminance. **a** PERG traces from one participant to a range of measured contrast levels in lights-ON (grey) versus lights-OFF (black) ambient light conditions. **b** Plot showing the relationship between mean luminance and stimulus contrast. The dual y-axis is shown with the scatter of individual P50 PT for each participant (circles) plotted against calculated contrast. The mean luminance at each contrast level are shown as lines for lights-ON and lights-OFF conditions

PERG waveforms recorded to 50’ check widths presented at a range of stimulus contrasts in a 30° field from one participant are shown against the photometrically measured stimulus contrast under each ambient lighting condition in Fig. 5a. This demonstrated a linear decrease in P50 amplitude with decreasing contrast. The P50 PT however was relatively constant to contrasts above 50% under each condition of ambient lighting. At the same calculated contrast for each ambient lighting the interpolated P50 PT was consistently earlier in lights-ON conditions compared to lights-OFF conditions (Fig. 5b).

### Pupil size, iris and skin colour

**Fig. 6:**
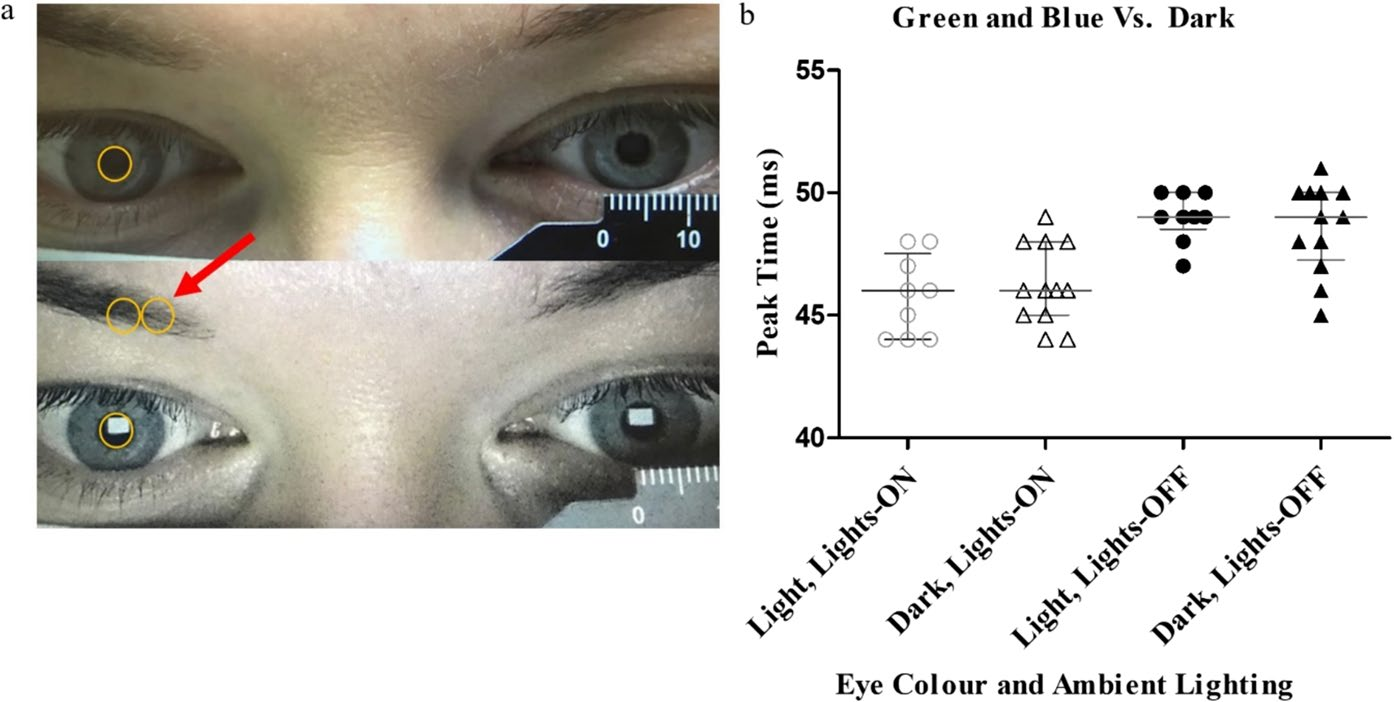
Pupil size and eye colour effects. **a** Pupil size is shown from one participant in lights-ON conditions (top panel) and lights-OFF conditions (lower panel). The orange circles were matched to pupil size in each condition and were directly comparable when overlaid (red arrow). **b** Scatter plot of P50 PT according to eye colour in each lighting condition (lights-ON on the left). The wider horizontal line represents the median and thinner horizontal line the 25^th^–75th centile

Pupil size was similar in lights-ON and lights-OFF ambient light conditions, as illustrated in Fig. 6a. Iris and skin colour varied across the group but was not associated with the magnitude of the P50 PT difference between ambient light conditions as illustrated in Fig. 6b.

### Effect of check width on the PERG in different ambient light conditions

**Fig. 7:**
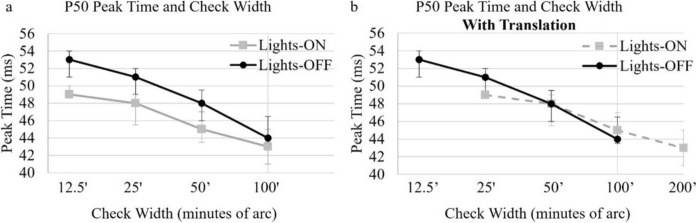
P50 PT plotted by check width in each ambient lighting condition. **a** Lights-OFF and lights-ON P50 PT to high contrast checks are plotted as a function of check width on a log scale (0.3 log units) and show similar increase of P50 PT to small check widths for each condition, but to the same check widths lights-ON P50 PT is consistently earlier than lights-OFF P50 PT. **b** A horizontal shift of the lights-ON P50 PT by one check width step on the abscissa (i.e. 0.3 log unit to the right on the *x*-axis) aligns the lights-ON and lights-OFF P50 PTs. The lights-OFF P50 PT to a 50’ check width is then comparable to the lights-ON P50 PT to a smaller 25’ check width. The median is plotted with interquartile range (25^th^–75th centiles)

In both ambient lighting conditions, the P50 PT became later as check width diminished in 0.3 log unit steps (Fig. 7a). A difference between lights-OFF and lights-ON P50 PT was greater to small check widths (Fig. 7a). It was noted that the lights-OFF P50 PT to a 50’ check was comparable to the lights-ON P50 PT to smaller 25’ check widths (Fig. 7b). A horizontal translation of the lights-ON P50 PT by one step right (i.e. increasing check width by 0.3 log unit) aligned lights-ON P50 PT with the lights-OFF P50 PT (Fig. 7b).

## Discussion

This study explored the effect of ambient lighting on the PERG. It found that the P50 PT was earlier recorded in lights-ON than lights-OFF ambient lighting conditions. In this study the lights-ON P50 PT from a 15° field was up to 6 ms earlier than the lights-OFF P50 PT within the same individual.

Our findings of an earlier P50 PT in lights-ON (117 cd.m^2^) compared to lights-OFF (4 cd.m^2^) ambient lighting conditions supports the observations of Bach and Schumacher [20], who compared P50 PT under three ambient lighting conditions: (1) P50 PT 46 ms lights-OFF (30 lx background; approximately 10 cd.m^2^), (2) 43 ms lights-ON (200 lx background; approximately 65 cd.m^2^), (3) 41 ms lights-ON (2300 lx background; approximately 750 cd.m^2^). These authors attributed an earlier lights-ON P50 PT to the effect of stray light reducing the stimulus contrast, as stimulus luminance was maintained at 50 cd.m^2^ for each condition [20]. Our photometric measurements demonstrated a 10.4% reduction of stimulus contrast and 8.5 cd.m^2^ increase in mean stimulus luminance in lights-ON ambient lighting using the highest software contrast setting (Table 1). This highlights that stimulus contrast and luminance should be measured under the same ambient lighting that will be used clinically. Some manufacturers perform photometric calibration in darkness to avoid light scatter from ambient lighting, but stimulus contrast will differ if recording PERGs in lights-ON conditions. This is particularly important given the recommendations in the ISCEV PERG Standard [9] which state that contrast should be maximal (close to 100%) and not less than 80%. Even with 100% software contrast under the lights-ON conditions of our study (117 cd.m^2^) the calculated stimulus contrast was close to this threshold (85.3%). The data in this study demonstrate that lights-OFF conditions provide the highest contrast and therefore are likely more reliable for inter-laboratory comparisons.

It is well documented that PERG P50 amplitude shows a linear decrease with reduction in stimulus contrast [16, 17, 21–23]. Studies have shown that the average PT of the transient pattern onset ERG does not vary with stimulus contrast within the SD of the group, with careful control of stimulus luminance [16]. An exploratory experiment of pattern reversal ERGs at different stimulus contrasts showed a stable P50 PT between 50 and 100% stimulus contrasts. This range encompasses the difference between lights-ON and lights-OFF stimulus contrasts measured as 85–96%. This small (11%) difference in stimulus contrast associated with stray light in lights-ON conditions therefore does not fully explain the earlier lights-ON P50 PT. P50 PT also does not appear to relate to natural pupil size which was similar in both ambient light conditions used in this study (Fig. 6a), as noted also by Bach and Schumacher [20].

As luminance increases RGC responses tend to become more transient with shorter latency. This has been associated with an increase in cone dominated phototransduction and faster inner-retinal circuitry [24]. Whilst this may contribute to an earlier P50 PT in the lights-ON conditions, the spatial properties of RGC receptive fields, such as receptive centre size and surround strength, are well recognised to vary with luminance [24]. P50 PT is known to alter with spatial frequency, becoming earlier to lower spatial frequency stimuli (i.e. larger check widths) [10–12, 25]. P50 PT also becomes earlier with increasing retinal eccentricity [26–30]. To better understand why ambient lighting alters P50 PT, we examined the effect of check width on PT in both ambient light conditions. In each condition, as previously reported [10–12, 25], we observed that larger check widths produce PERGs with earlier P50 PTs compared to smaller check widths from the same field size. Whilst the P50 is a ‘macular cone driven’ response, it is thought that around 70% of this response amplitude reflects activity of RGCs [1]. The RGC receptive field centre separation increases with increasing retinal eccentricity from the central field. Consequently, a PERG from more peripheral retina is predicted to show higher amplitudes to large check widths. Whilst in the central visual field the PERG is expected to be bigger to small check widths where the RGC receptive fields are smaller and closer together. This is analogous to the scaling of visual acuity with eccentricity [31, 32].

An increase in RGC receptive field centres occurs under mesopic ambient lighting [10, 11, 24, 25]. In lights-OFF conditions the spatial sensitivity of the PERG is speculated to shift to lower spatial frequencies compared to that observed in lights-ON conditions. Earlier work exploring the spatial sensitivity of the PERG showed 0.75 (45’) and 1 degree (60’) check widths produced the largest PERG amplitude in 15° and 30° field sizes and are likely optimised for RGC receptive field centre separation. This study did not specify ambient lighting conditions [33]. Our study of PERGs to different spatial frequencies presented in a 30° field showed that the P50 PT to a 25’ check width under lights-ON conditions is the same as the P50 PT to a 50’ check width under lights-OFF conditions. In other words, the 50’ check width acts effectively as a ‘large’ check width under lights-ON conditions and produces an earlier PT than it does under lights-OFF conditions (Fig. 7).

The stimulus surround of the 15° and 30° square fields was matched to the mean luminance of the pattern stimulus in this study. The stimulus mean luminance varied by 8.5 cd.m^2^ between lights-ON and lights-OFF conditions. The peripheral ambient lighting beyond the display monitor differed by ~ 100 cd.m^2^, (from 4 to 117 cd.m^2^) between lights-ON and lights-OFF conditions. It is more likely that adaptation to this ambient lighting, rather than a much smaller change in mean stimulus luminance, influenced the P50 PT. Peripheral field mesopic adaptation mechanisms suggest rod–cone interactions cooperate to balance the need for sensitivity, gain and acuity [34, 35]. These interactions begin with luminance changes at rod–cone gap junctions, to horizontal cell feedback and recruitment of amacrine cell circuitry [24].

Although our study showed that the group lights-ON P50 PT to 25’ check widths is comparable to lights-OFF P50 PT to standard (50’) checks, the reason for the inter-individual variation of P50 PT is not yet explained. There was not a clear relationship with age nor ocular or skin pigment, indeed the two participants with maximal PT changes had blue eyes and fair skin aged 30 years and hazel eyes and brown skin aged 32 years [36].

Clinically, these findings have implications for inter-laboratory comparison of the PERG and for combining reference data. Background ambient lighting is a variable that should be stated in reports and ideally standardised across laboratories. This is particularly important if monitoring patients with optic neuropathy, when a shortening of PT may be interpreted as an additional signal of RGC dysfunction. The PERG Standard update (2024) now includes a need to specify ambient lighting for reports and reference data [9].

One limitation of this work is that it was performed in healthy participants. Future studies may wish to explore whether these alterations have any consequence in abnormal states, for example in diseases affecting the macular or RGCs. Whilst we expect the relative sensitivity of the PERG may be the same for both lights-ON and lights-OFF conditions, it could be hypothesised that diseases which may affect RGC receptive field density may differentially affect the spatial sensitivity of the PERG, therefore influencing the PERG differently.

## Conclusion

The PERG P50 PT is earlier in lights-ON than lights-OFF ambient light conditions to the same check width. The lights-OFF P50 PT to 50’ check width is the same as the lights-ON P50 PT to smaller 25’ check widths. Changes with check width and ambient lighting may be due to adaptive retinal interactions altering RGC spatial sensitivity. Consistent ambient lighting is essential for clinical recordings, and these findings support the specification of ambient lighting in clinical reports for future inter laboratory comparison of data.
